# Kernel Nutrient Composition and Antioxidant Ability of *Corylus* spp. in China

**DOI:** 10.3389/fpls.2021.690966

**Published:** 2021-06-23

**Authors:** Jiangzhao Jiang, Lisong Liang, Qinghua Ma, Tiantian Zhao

**Affiliations:** ^1^Research Institute of Forestry, Chinese Academy of Forestry, Beijing, China; ^2^State Key Laboratory of Tree Genetic and Breeding, Beijing, China; ^3^Hazelnut Engineering and Technical Research Center of the State Forestry and Grassland Administration, Beijing, China; ^4^National Innovation Alliance of Hazelnut Industry, Beijing, China; ^5^Nanchong Vocational and Technical College, Nanchong, China

**Keywords:** antioxidant, hazelnut in China, fat acid, amino acids, sugar, tocopherol, total phenol, total flavonoid

## Abstract

Hazelnut (*Corylus*) is an important woody oil tree species in economic forests. China, as one of the original countries of native *Corylus* species, had 8 species and 2 varieties. However, little information is available on the hazelnut nutritional quality of these Chinese *Corylus* species. In this study, four main wild *Corylus* species (*C. heterophylla* Fisch., *C. mandshurica* Maxim., *C. kweichowensis* Hu., and C. *yunnanensis* Franch.) originating in China and one main cultivar of hybrid hazelnut (*Corylus heterophylla* Fisch. × *C. avellana* L.) cv. ‘Dawei’ from China were used to analyze the basic nutritional composition (content of oil, fatty acid, protein, saccharide, aminao acid, vitamin C, tocopherol, total phenols, and total flavonoids) and antioxidant ability. The results showed that oil content ranged from 52.97 to 60.88 g/100 g DW and highly unsaturated fatty acid (UFA) content was over 91%. Oleic was the most dominant UFA in these hazelnut kernels, and the relative content was ranging from 71.32 to 85.19%. Compared with other four hazelnut kernels, *C. heterophylla* Fisch. was the lowest oil content of hazelnut with lower oleic acid content and higher linoleic acid content, obviously. The total protein content ranged from 13.15 to 18.35 g/100 g DW, and all amino acids were detected as hydrate amino acids, but Tryptophan, an essential amino acid, was not detected as free amino acid in these hazelnut kernels. Kernel of *C. heterophylla* Fisch. was with the highest content of protein and amino acid. Saccharose was the most essential and abundant disaccharide in the hazelnut kernels. C. *mandshurica* Maxim. was the highest saccharide content among these hazelnut kernels. α-tocopherol was the main type of tocopherol found in the hazelnut kernels. Wild hazelnut kernels generally had higher bioactivity substance content (vitamin C, total tocopherol, total phenol and total flavonoid) and antioxidant capacity. Compared to the four wild hazelnut kernels, the hybrid hazelnut cv. ‘Dawei’ had higher content of oil, oleic acid, α-tocopherol and sugar. Overall, there were great differences in the nutritional composition of different hazelnut species. Wild species are a good source of breeding materials because of their own characteristics in nutrition composition, and the hybrid hazelnut cv. ‘Dawei’ with good quality has the value of commercial promotion.

## Introduction

Hazelnut (*Corylus*), an important economic forest and nut tree, generally grows in temperate climate zones with a relatively high humidity and a high rainfall rate (Kole, 2011). China, as one of the original countries of native *Corylus* species, had 8 species and 2 varieties, including *Corylus heterophylla* Fisch., *Corylus mandshurica* Maxim., *Corylus kweichowensis* Hu., *Corylus chinensis* Franch., *Corylus fargesii* C. K. Schneid., *Corylus yunnanensis* (Franch.) A. Camus, *Corylus ferox* Wall., *Corylus wangii* Hu., *Corylus kweichowensis*var. *brevipes* W. J. Liang, and the *Corylus ferox* Wall. var. *thibetica* (Batalin) Franch. The distribution of these resources ranges from 24°31’N to 51°42’N covering 24 provinces in China (Zhang et al., 2005). Among these wild *Corylus* species in China, *C. heterophylla* has been the most valuable and widely utilized until recently and is distributed mainly over the north and northeast of China. In recent years, more and more local people have started to cultivate and manage *C. heterophylla*, with the yield and production continuing to grow sustainably. *C. mandshurica*, which is distributed at higher altitudes and has a very fragrant flavor, *C. kweichowensis*, which grows in the middle and southern regions of China, and *C. yunnanensis*, a high shrub or a small arbor tree, which is distributed at higher altitudes from 2,000 to 3,700 m in the southwest of China, are the other three wild species that can be focused on due to the ease of collection relative to the rest of the wild *Corylus* species.

The hybrid hazelnut (*C. heterophylla* Fisch. × *C. avellana* L.) is a new hazelnut species obtained by crossbreeding using selected *C. heterophylla* Fisch. from northeast China as the female parent and mixed pollen of several European hazelnut (*C. avellana* L.) seedlings introduced from Italy as the male parent since 1980’s (Liang et al., 2012). Approximately 15 varieties of hybrid hazelnut were selected at the end of the twentieth century and were released gradually from the year 2000 to 2010. Cv. ‘Dawei’, as the main cultivar of the hybrid hazelnut (*C. heterophylla* Fisch. × *C. avellana* L.), has the properties of large size, high yield and good climate adaptability and is planted widely in the north, northeast, northwest and even central regions in China. To date, the total cultivated area of hybrid hazelnut (*C. heterophylla* Fisch. × *C. avellana* L.) in China is approximately 50,000 ha, It accounts for more than half of the current cultivated area.

Among nut species, hazelnut plays a major role in human nutrition and health because of its special composition of fats (approximately 60%), most of which are monounsaturated fatty acids (MUFA) (mainly oleic acid), protein, carbohydrate, dietary fiber, vitamins (vitamin E), minerals, phytosterols (mainly β-sitosterol), squalene and antioxidant phenols (Amaral et al., 2006; Alasalvar et al., 2003, 2006, 2010; Rio et al., 2011; Bacchetta et al., 2013; Cosmulescu et al., 2013; Ciarmiello et al., 2014). More and more studies have shown that nuts may play a role in the prevention of chronic age-related diseases, such as Alzheimer’s disease (Gorji et al., 2017). Frequent nut consumption has been associated with better metabolic status and a significant decreased risk of cardiovascular disease and mortality (especially that due to cardiovascular-related causes); it is also associated with reduced risks of certain types of cancers, such as colorectal, endometrial, and pancreatic neoplasms (Grosso and Estruch, 2015); it also effects on human innate response, such as antiinflammatory (Giulia et al., 2018). *Corylus avellana* oil was found to be effective in the treatment of polycystic ovary syndrome, via the regulation of gonadotropins, steroids and serum lipid parameters, and it possesses antioxidant activity (Demirel et al., 2016). Hazelnut-enriched diets may exert antiatherogenic effects by improving endothelial function, preventing LDL oxidation, and activating inflammatory markers, in addition to their lipid and lipoprotein-lowering effects (Orem et al., 2013; Santi et al., 2017; Benassi et al., 2019, 2021).

Previous research has shown that the nutrient composition and content of hazelnut in different varieties or cultivars are very different (Köksal et al., 2006; Matthäus and Özcan, 2012; Tian et al., 2012). For example, the fat content ranges from 50.6 to 66.4%, the saturated fatty acid (SFA) content from 3.7 to 15.6%, the MUFA content from 40.1 to 84.7%, the polyunsaturated fatty acid (PUFA) content from 1.5 to 16.4%, and the total unsaturated fatty acid (UFA) content from 70.0 to 93.93%, but most hazelnut kernels are similar in their main fatty acid composition. The protein content ranges from 12.6 to 25.9 g/100 g, the carbohydrate content approximately 6.5–24.0 g/100 g and the vitamin E content approximately 15–20 mg/100 g (Oliveira et al., 2008; Alasalvar et al., 2010; Ma and Liu, 2011; Chai et al., 2012).

China, as an important distribution area of *Corylus* in the world, has abundant resources of *Corylus*. A better understanding of its genetic diversity and its distribution and its biochemical characteristics, is a prerequisite to improving breeding programs, enhancing the competiveness of the hazelnut production, but *Corylus* genetic wild resources are highly underutilized (Bacchetta et al., 2015). Investigation of the nutrient composition and antioxidant capacity of *Corylus* cultivars and species originating from China is a very important part of *Corylus* plant research worldwide. To our knowledge, few reports of basic information of Chinese wild hazelnut kernels and hybrid hazelnut (*C. heterophylla* Fisch. × *C. avellana* L.) kernels can be found. However, research on hazelnut breeding in China is just beginning, and many problems, especially nut quality characteristics, are hampering advancement. Thus, analysis and comparison of the characteristics of the nutrient composition and antioxidant abilities of wild hazelnut kernels originating from China as well as Chinese hybrid hazelnut kernels are important to provide information for breeding and resource assessments of *Corylus*.

## Materials and Methods

### Materials Collections

Four Chinese wild species of hazelnut kernels, *Corylus heterophylla* Fisch. (the Chinese name is the Ping hazelnut), *Corylus mandshurica* Maxim. (the Chinese name is the Mao hazelnut), *Corylus kweichowensis* Hu. (the Chinese name is the Chuan hazelnut) and *Corylus yunnanensis* (Franch.) A. Camus (the Chinese name is Dian hazelnut), were obtained from their main distribution areas in Northeast China, North China, the Dabie Mountain area and Northwest China, respectively. One seven-year-old Chinese main cultivar of the hybrid hazelnut (*C. heterophylla* Fisch. × *C. avellana* L.) cv. ‘Dawei’ was collected from the plantation of the Research Institute of Forestry, Chinese Academy of Forestry in Beijing. The period of collecting nuts, site and main soil characteristics are shown in Table 1. A total of 2.5 kg of each sample of hazelnut was collected for analysis.

**TABLE 1:** Nuts collecting period, sites and main soil characteristics of the parcel.

**Species/cultivars**	**Time**	**Collection sites**	**Soil**	**Geographic coordinates**	**Nut maturity**
*C. kweichowensis*	October, 2017	Huoshan County, Anhui Province	Yellow brown soil	N31°03′—31°33′E115°52′—116°32′	Natural maturity
*C. mandshurica*	September, 2017	Weichang County, Hebei Province	Brown soil	N41°35′∼42°40′E116°32′∼118°14′	Natural maturity
*C. heterophylla*	September, 2017	Tieling City, Liaoning Province	Brown soil	N41°59′∼42°33′E123°28′∼124°33′	Natural maturity
*C. yunnanensis*	October, 2017	Zhaotong City, Yunnan Province	Red soil	N26°34′—28°40′E102°52′—105°19′	Natural maturity
cv. ‘Dawei’	August, 2017	Yanqing District of Beijing	Brown soil	N40°16′∼40°47′E115°44′∼116°34′	Natural maturity

### Sample Preparation

Before the shells were removed, the water content of the fresh hazelnut kernels was decreased to 6.20% after 10 days of air-drying (average of wind velocity, ambient air temperature, relative humidity and sunshine duration; 3.1 m/s, 16°C, 70%, 6.5 h, respectively). The dry matter content in the kernel was determined after drying at 80 ± 2°C until a constant weight was achieved and then the kernels were smashed to obtain a kernel sample from each hazelnut. A Soxhlet extractor (extracted with ether for 8 h at 50°C) was used to extract the oil of the kernel to simultaneously obtain the oil sample and the defatted flour sample. Each defatted flour sample was collected by milling and sifting at 150 μm.

### Oil Content and Fatty Acid Composition

Oil content was determined by extracting a known weight of the sample (5 g) with ether (50°C) for 8 h using the Soxhlet extractor. Fatty acid composition was determined by gas chromatography (GC) with a flame ionization detection (FID) capillary column, based on the following procedure: 100 μL of oil was used for fatty acid methylation, with 1 mL of methyl esterification reagent (0.5 mol/L KOH-CH_3_OH solution) and 2 mL of ether, for at least 3 h at room temperature. Then, 5 mL of deionized water was added to obtain phase separation, and the fatty acid methyl ester (FAME) was recovered with 5 mL of diethyl ester by shaking in a vortex. The upper phase was passed through a micro-column of anhydrous sodium sulfate to eliminate the water, and the sample was recovered in a vial with Teflon; before injection, the sample was filtered with a 0.2 μm nylon filter from Millipore. The fatty acid profile was analyzed with an Agilent GC 7890A instrument equipped with a split/splitless injector, an FID and a AB-FFAP column (30 m × 0.25 mm × 0.25 μm). The oven temperature program was as follows: the initial temperature of the column was 50°C and was held for 1 min; then, a 15°C/min ramp to 200°C was held for 45 min. The carrier gas (nitrogen) pressure was 20 PSI, and the FID detector temperature 270°C. Split injection (20:1) was carried out at 250°C. For each analysis, 1 mL of the sample was injected in GC. The results were recorded and processed using the Agilent ChemStation GC system and were expressed as the relative percentages of each fatty acid, calculated by internal normalization of the chromatographic peak area. Fatty acids were identified by comparing the relative retention times of the FAME peaks from the samples with those of the standards (Seyhan et al., 2007; Oliveira et al., 2008). Oil content is presented as g/100 g DW, and fatty acid relative content is expressed as %.

### Protein Content

The total protein content (TPrC) was determined by the micro Kjeldahl method using 0.5 g of the defatted flour sample. TPrC was calculated as total N × 5.3 (Cunniff et al., 2000). Soluble protein content (SPrC) was determined by the Coomassie brilliant blue G250 staining method. Approximately 1 g of each kernel sample was dissolved in 50 mL of deionized water and was then ultrasonic extracted at 40°C for 30 min. The extraction solution was filtered with filter paper, and then the filtrate was collected. Next, 5 μL of filtrate was the added to a 96-well microplate, followed by the addition of 195 μL of Coomassie brilliant blue G250 reagent; the plate was then kept at room temperature for 5 min. The absorbance of the mixture was measured at 595 nm using an ELISA plate reader, and the concentrations were compared to a standard curve based on a prepared bovine serum albumin (BSA) standard solution (0, 200, 400, 600, 800 and 1,000 μg/mL). SPrC is expressed as g/100g DW.

### Amino Acid Content

Amino acid content was determined using reversed-phase high-performance liquid chromatography fluorescence detection (RP-HPLC) (Waters 2695). RP-HPLC was performed with an AccQ Taq aa column (3.9 mm × 150 mm) and a 2475 detector. The excitation wavelength (Ex) and the emission wavelength (Em) of the fluorescence detector were 250 and 395 nm, respectively. Two different solvents, namely, solvent (A), a buffer solution of 140 mmol/L sodium acetate (17 mmol/L triethylamine, pH 4.95) and solvent (B), a buffer solution of 60% acetonitrile water solution, were gradually used at 1.0 mL/min flow rate. The free aminoacid content is presented as mg/100 g DW and the hydrolyzed aminoacid content is presented as g/100 g DW.

### Composition and Content of Sugar

Sugar composition was determined using high performance liquid chromatography (HPLC). A total of 0.5 g of each kernel sample was dissolved in 20 mL of deionized water and incubated in a boiling water bath for 30 min. The samples were then filtered to collect the supernatant as the sample solution. Next, 20 μL of the sample solution was used to determine the sugar composition. The content of polysaccharide, tetrasaccharide, trisaccharide, disaccharide, fructose, glucose and mannitol were analyzed with a Waters 2695 instrument equipped with a 2414 Refractive Index Detector (Yuan et al., 2018) and sugar-pak-1 column (6.5 mm × 30 m); deionized water was the moving phase, with a 0.6 mL/min flow rate and a column temperature of 70°C. Glucan (relative molecular weight 10,000), stachyose tetrahydrate, raffinose and maltose were used as standards for polysaccharide, tetrasaccharide, trisaccharide and disaccharide, respectively. The content of xylose, galactose and saccharose were analyzed with a Waters 2695 instrument equipped with a 2414 Refractive Index Detector (Yuan et al., 2018) and NH_2_ column (4.6 mm × 250 mm); 78% acetonitrile was used as a moving phase, with a 1.0 mL/min flow rate. All the values are presented as g/100 g DW.

### Vitamin C Content

Vitamin C content was determined using the Molybdenum blue colorimetry method. A total of 2.0 g of each kernel sample was dissolved in 10 mL of an oxalic acid—EDTA solution and then was ultrasonically extracted at room temperature for 30 min. The extracting solution was then centrifuged at 3,000 × g for 10 min to obtain the supernatant. Then, 5 mL of supernatant was added to a glass tube, followed by the addition of 0.5 mL of a metaphosphoric acid-acetic acid solution, 1.0 mL of 5% sulfuric acid and 2.0 mL of ammonium molybdate; the sample was then kept at 30°C for 15 min after mixing. The absorbance of the mixture was measured at 760 nm using an ultraviolet—visible spectrophotometer, and absorbance was then compared to a standard curve based on a prepared ascorbic acid standard solution (0, 8, 16, 24, 32, and 40 μg/mL). Vitamin C content is expressed as mg/100 g DW.

### Tocopherol Composition

α-, β-, γ-, and δ- tocopherols were measured by RP-HPLC and a UV detector at 284 nm based on the method by Kornsteiner et al. (2006). For the determination of tocopherols, 250 mg of oil was dissolved in 0.2 mL of diethyl ether, and then 20 μL of the oil solution was used for HPLC. The tocopherol composition was then analyzed with a Waters 2695 instrument equipped with an ultraviolet detector (wavelength of measurement was 284 nm) and a Sunfire C18 column (4.6 × 250 mm × 5 μm). Methyl alcohol was the moving phase, with a 1.2 mL/min flow rate. All the values are presented as mg/100 g oil.

### Content of Total Phenolic (TPC) and Total Flavonoid (TFC)

#### Sample Extraction

A total of 1.0 g of each kernel sample was dissolved in 40 mL of 60% ethyl alcohol solution (containing 0.024% HCL) and then placed at 75°C in a water bath for 50 min after mixing. The mixture was then centrifuged at 3,000 r/min for 20 min, and the supernatant was collected.

#### TPC Measurement

TPC was measured by the Folin–Ciocalteu method with slight modifications (Bolling et al., 2010). A total of 25 μL of supernatant was added to 50 μL of Folin–Ciocalteu reagent and 100 μL of a 12% sodium carbonate solution, and then the sample was placed in the dark at room temperature for 2 h. The absorbance of the mixture was measured at 765 nm in an ultraviolet—visible spectrophotometer and was compared to a standard curve based on a prepared gallic acid (GAE) standard (25, 50, 75, 100, 125, and 150 μg/mL). TPC is expressed as mg GAE/100 g DW.

#### TFC Measurement

TFC was determined using a colorimetric method with slight modifications (Balta et al., 2006). A total of 40 μL of supernatant was added to 60 μL of a 60% ethyl alcohol solution and 6 μL of a sodium nitrite solution, was vortexed and was then placed at room temperature for 6 min. Next, 6 μL of 10% Al (NO_3_)_3_ was added; after 6 min, 80 μL of NaOH was added, and the sample was placed at room temperature for 12 min. The absorbance of the mixture was measured at 510 nm by an ultraviolet and visible spectrophotometer and was compared to a standard curve based on a prepared rutin standard (40, 80, 160, 240, 320, and 400 μg/mL). TFC is expressed as mg/100 g DW.

### ABTS and DPPH Free Radical Scavenging Rate

#### Sample Extraction

A total of 0.25 g of each kernel sample and defatted flour sample was dissolved in 10 mL of a 60% ethyl alcohol solution (containing 0.024% HCL) and then ultrasonically extracted at 50°C for 30 min. The mixtures were filtered to collect the supernatant as the sample solutions.

#### ABTS Free Radical Scavenging Rate

The ABTS free radical scavenging rate was measured by the Oliveira method with slight modifications (Oliveira et al., 2008). A total of 50 μL of the supernatant from each kernel sample, defatted flour sample and pure oil sample was added to a 96-well microplate, followed by the addition of 150 μL of the ABTS reagent (mixed 7 mmol/L ABTS solution and 2.5 mmol/L potassium persulfate solution together in a 1:1 proportion then diluted 5 times), and the plate was then placed in the dark at room temperature for 30 min. Then, 50 μL of the two supernatants and oil were added to 150 μL of a 60% ethyl alcohol solution as the sample blanks, and the 60% ethyl alcohol solution was added to 150 μL of the ABTS reagent as the reagent blank. The absorbance of the mixtures was measured at 734 nm by an ELISA plate reader, and the ABTS free radical scavenging rate was calculated according to the following equation:

ABTSradicalscavengingrate(%)=(1-A⁢1-A⁢2A⁢ 0)×100

where A1 is the absorbance of the sample solution, A2 is the absorbance of the sample blank and A0 is the absorbance of the reagent blank.

#### DPPH Free Radical Scavenging Rate

The DPPH free radical scavenging rate was measured by the Oliveira method with slight modifications (Oliveira et al., 2008). A total of 50 μL of the supernatant of each kernel sample, defatted flour sample and pure oil sample was added to a 96-well microplate, followed by the addition of 150 μL of a 200 μmol/L DPPH solution; then, the plate was placed in the dark at room temperature for 30 min. Next, 50 μL of two supernatants and oil were added to 150 μL of a 60% ethyl alcohol solution as the sample blanks, and 60% ethyl alcohol solution was added to 150 μL of a 200 μmol/L DPPH solution as the reagent blank. The absorbance of the mixtures was measured at 517 nm using an ELISA plate reader, and the DPPH free radical scavenging rate was calculated according to the following equation:

DPPHradicalscavengingrate(%)=(1-A⁢1-A⁢2A⁢ 0)×100

where A1 is the absorbance of the sample solution, A2 is the absorbance of the sample blank and A0 is the absorbance of the reagent blank.

### Statistical Analysis

The data are presented as the mean ± SE (standard error) of three independent experiments, with three replicates for each. The statistical analysis was performed using SPSS 13.0 software (SPSS Company, Chicago, IL, United States). Differences between the different species and cultivars were analyzed by one-way ANOVA, taking *P* < 0.05 as the level of significance according to Duncan’s multiple range test. The correlation between the free radical scavenging rate and nutrients was analyzed by Pearson correlation coefficient (test of significance, using two-tailed distribution).

## Results and Discussion

### Oil and Fatty Acid Contents

Oil is the predominant component with high value in nuts. Nut oil is mostly unsaturated, and UFAs have been associated with beneficial effects. In general, oil content is largely different among different hazelnut species and varieties and even in the same cultivar produced in different areas. Matthäus reported (Matthäus and Özcan, 2012) that the total oil content of five hazelnut samples collected from Turkey and Germany ranged widely from 8.1 to 64.1%, however, the total oil content of the seventeen hazelnut varieties from Turkey was 56.07–68.52%, and in three cultivars and in two agricultural practices of Oregon hazelnut kernels were 57–65 g/100 g in other reports (Köksal et al., 2006; Wang et al., 2018). The oil content of different varieties of hybrid hazelnut kernels (*C. heterophylla* Fisch. × *C. avellana* L.) from China was 53.80–63.33%, and *C. heterophylla* has an average content of 57.95% (Tian et al., 2012). In this research, the oil content of the five different hazelnut kernels ranged from 50.23 to 60.88 g/100 g DW, as shown in Table 2. *C. heterophylla* had the lowest oil content among the four wild species, with the same levels as *C. kweichowensis*. *C. mandshurica*, and *C. yunnanensis* were the wild species with the highest oil content. The hybrid hazelnut cv. ‘Dawei’ had the highest oil content among these five hazelnut kernel samples, which was obviously higher than that of the female parent *C. heterophylla*, and was similar in level to the oil content of the two wild species *C. mandshurica* and *C. yunnanensis*.

**TABLE 2:** Oil content and Fatty acid composition of hazelnut kernels from different Chinese hazelnut species and cultivars.

**Species/**	**Oil content**	**Fatty acid content (%)**													
**cultivars**	**(g/100 g DW)**														
		**C_16:0_**	**C_16:1_**	**C_18:0_**	**C_18:1_**	**C_18:2_**	**C_18:3_**	**C_20:0_**	**C_20:1_**	**SFA**	**UFA**	**MUFA**	**PUFA**	**MUFA/**	**UFA/**
														**PUFA**	**SFA**
*C. kweichowensis*	52.97 ± 0.42c	3.03 ± 0.04d	0.14 ± 0.01cd	1.15 ± 0.03d	84.89 ± 0.09a	10.17 ± 0.08c	0.12 ± 0.01c	0.08 ± 0.00b	0.31 ± 0.01a	4.26 ± 0.02d	95.63 ± 0.02b	85.34 ± 0.10a	10.29 ± 0.08c	8.29	22.45
*C. mandshurica*	58.40 ± 0.54b	5.85 ± 0.04a	0.28 ± 0.01a	2.60 ± 0.01b	80.19 ± 0.12c	10.47 ± 0.12c	0.10 ± 0.00c	0.18 ± 0.01a	0.20 ± 0.01c	8.63 ± 0.02a	91.24 ± 0.01e	80.67 ± 0.11b	10.57 ± 0.11c	7.63	10.57
*C. heterophylla*	50.23 ± 0.26d	2.06 ± 0.02e	0.11 ± 0.01d	0.95 ± 0.04e	71.32 ± 0.23d	24.36 ± 0.23a	0.29 ± 0.00a	0.08 ± 0.01b	0.27 ± 0.00b	3.08 ± 0.01e	96.36 ± 0.46a	71.70 ± 0.24c	24.66 ± 0.23a	2.91	31.29
*C. yunnanensis*	59.75 ± 0.51a	4.49 ± 0.02b	0.15 ± 0.01c	2.77 ± 0.04a	80.78 ± 0.13b	11.21 ± 0.12b	0.26 ± 0.01b	0.09 ± 0.01b	0.13 ± 0.01d	7.35 ± 0.02b	92.52 ± 0.02d	81.05 ± 0.13b	11.47 ± 0.11b	7.07	12.59
cv. ‘Dawei’	60.88 ± 0.33a	3.90 ± 0.02c	0.20 ± 0.01b	1.51 ± 0.03c	85.19 ± 0.06a	8.58 ± 0.07d	0.10 ± 0.01c	0.10 ± 0.01b	0.26 ± 0.01b	5.51 ± 0.04c	94.33 ± 0.02c	85.65 ± 0.06a	8.68 ± 0.07d	9.87	17.12

*Data are means ± standard error (SE), n = 3. Values in the same column with different letters are significantly different (p < 0.05). SFA, saturated fatty acid; UFA, unsaturated fatty acid; MUFA, monounsaturated fatty acid; PUFA, polyunsaturated fatty acid.*

Fatty acids are the most important components of hazelnut kernel oil, including SFAs and UFAs. A number of studies suggest that nuts confer significant protective effects against depression, mild cognitive disorders and Alzheimer’s disease (Gorji et al., 2017). The underlying mechanisms appear to include antioxidant and anti-inflammatory actions, particularly related to their mono- and polyunsaturated fatty acids. MUFAs have been demonstrated to improve pancreatic beta-cell function and to regulate postprandial glycemia and insulin sensitivity. PUFAs may act on the central nervous system, protecting neuronal and cell-signaling function and maintenance (Grosso and Estruch, 2015). Eight main fatty acids were detected from these hazelnut samples, as shown in Table 2. The oleic acid content, as the highest of the fatty acids, widely ranged from 71.32 to 85.19% among these five different hazelnut kernels. Linoleic acid, whose content ranged from 8.58 to 24.36%, was the second main composition of fatty acid, although it was remarkably lower than oleic acid content. Palmitic acid and stearic acid were the two dominant saturated fatty acids in these hazelnut samples. The comparison of these results showed that there were very different contents of each fatty acid among the four different wild species of hazelnut. *C. kweichowensis* had the highest content of oleic acid at 84.89%, which was higher than several Turkish hazelnut varieties that Köksal reported (Köksal et al., 2006). *C. mandshurica* had the lowest oleic acid content at 71.32%. *C. heterophylla* and *C. yunnanensis* had equivalent levels for oleic acid content, at approximately 80%. In terms of linoleic acid content, *C. heterophylla* had the highest content of 24.36%, which was obviously higher than the other three wild species of hazelnut kernel. The highest palmitic acid content of the wild hazelnut kernel was *C. mandshurica*, with more than twice that of *C. heterophylla*, at 2.06%. Compared to the four wild hazelnut kernels, the hybrid hazelnut cv. ‘Dawei’ had the highest content of oleic acid (85.19%), the lowest content of linoleic acid (8.58%) and an average level of palmitic acid (3.90%) (Table 2). As the new hazelnut species, the fatty acid profile of the hybrid hazelnut cv. ‘Dawei’ changed profoundly, with the oleic acid content increasing and the linoleic acid content decreasing drastically, compared to *C. heterophylla* and the other Chinese wild hazelnut species.

Further analysis indicated that hazelnut kernels were the nuts with the highest UFA content (over 91%) and the lowest SFA content (below 9%, Table 2). In particular, *C. kweichowensis* and *C. heterophylla* had higher contents of total UFA at more than 95% and had a lower total SFA at less than 4.5% among the four hazelnut species. The contents of SFA and UFA in the hybrid hazelnut cv. ‘Dawei’ were all at average levels. Over 70% MUFA and less than 25% PUFA made up the UFA content in each variety of hazelnut. The ratios of MUFA / PUFA and UFA / SFA among the four wild species of hazelnut kernels were 2.91–8.29 and 10.57–31.29, respectively, while the hybrid hazelnut cv. ‘Dawei’ had the highest MUFA/PUFA ratio of 9.87 and an average UFA / SFA ratio of 17.12 (Table 2), which was higher than that of any varieties of Turkish hazelnut reported by Köksal et al. (2006). Overall, these wild species of hazelnut kernels as well as the hybrid hazelnut cv. ‘Dawei’ had profile characteristics of higher UFA content and lower SFA content.

### Protein Content

**FIGURE 1:**
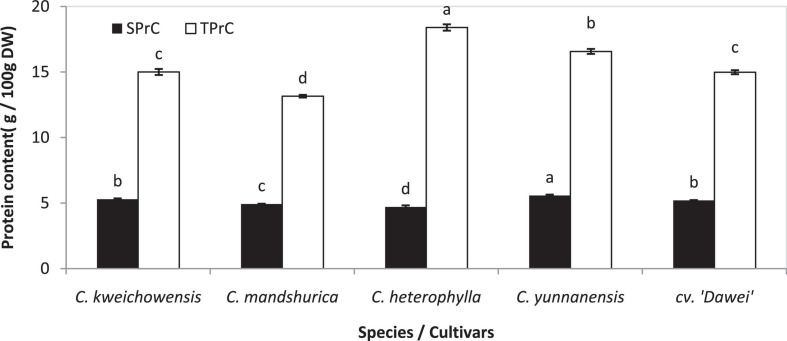
Total protein and soluble protein content of hazelnut kernels from different Chinese hazelnut species and cultivars. SPrC, soluble protein content; TPrC, total protein content. Data are presented as means ± SE (standard error) of three independent experiments with three replicates for each. Values followed by a different letter were significantly different according to Duncan’s multiple range test at *P <* 0.05.

Proteins perform various functions in food systems and are an important component in nuts. Recent research has indicated that the effects of roasting on the functional properties of defatted hazelnut flours, such as oil absorption, water absorption capacity, emulsion and foaming properties, were related to proteins of the hazelnut to a large extent (Turan et al., 2015). The protein content of the hazelnut kernel varied slightly in different areas in several different reports, at approximately 12.6–25.9 and 14.5–18.2 g/100 g (Peh et al., 2016; Wang et al., 2018). In this research, all of the hazelnut varieties had a total protein content (TPrC) of 13.15 to 18.39 g/100 g DW, and *C. heterophylla* had the highest level among these five hazelnut kernel samples. The TPrC of the hybrid hazelnut cv. ‘Dawei’ was remarkably lower than that of its female parent, *C. heterophylla* (*p* < 0.05) (Figure 1). The TPrC results of Chinese hazelnut kernels were in accordance with those of previously published hazelnut data (Köksal et al., 2006; Turan et al., 2015; Wang et al., 2018). Previous research about the nutrients of 33 hybrid hazelnut (*Corylus heterophylla* Fisch. × *C. avellana* L.) kernels varieties and *C. heterophylla* reported that the soluble protein content (SPrC) of these varieties were 29.22 to 68.57 mg/g and an average of 56.91%, respectively (Tian et al., 2012). The SPrC in these five hazelnut kernel samples was approximately 4.70–5.58 g/100 g DW, comprising approximately 25.6–37.5% of the total protein content (Figure 1). This result was consistent with that of Tian’s report. Compared to the female parent *C. heterophylla*, the TPrC decreased, but the SPrC increased significantly in kernel of hybrid hazelnut cv. ‘Dawei’ (*p* < 0.05).

### Amino Acid Content

In the present study, the free amino acid (FAA) composition and the content of the hazelnut kernel is given in Table 3. Fourteen to fifteen amino acids were detected in each hazelnut sample, including six to seven essential amino acids (EAA) and eight to nine non-essential amino acids (NEAA), and the differences were obvious for each of them. The total EAA content ranged from 4.71 to 15.26 mg/100 g DW, and *C. kweichowensis* and *C. yunnanensis* had significantly higher total EAA content than those of the other three hazelnut kernels (*p* < 0.05). Threonine (Thr) was the EAA with the highest content in *C. kweichowensis* and *C. yunnanensis* kernels, at 11.27 and 9.00 mg/100 g DW, respectively, which was obviously higher than those of the other three hazelnut kernel samples. Methionine (Met) was only detected in the FAA of *C. yunnanensis*, and the content was very low, at 0.26 mg/100 g DW. Tryptophan (Trp) was not detected in the FAA of all the hazelnut kernel samples. The total NEAA content was 14.07–26.15 mg/100 g DW and that of *C. heterophylla* was the highest. All the varieties of hazelnut were relatively high in Glutamic acid (Glu) (Taş and Gökmen, 2015) content 4.34–8.15 mg/100 g DW. The hybrid hazelnut cv. ‘Dawei’ had the highest Glu content, which was obviously higher than four wild hazelnut species. The results of the total FAA content showed that *C. kweichowensis*, *C. heterophylla*, and *C. yunnanensis* were at similarly high levels, ranging from 30.86 to 33.29 mg/100 g DW. The lowest level of FAA content among the four wild hazelnut species was *C. mandshurica*, which was almost forty percent lower than that of the highest, *C. kweichowensis*. The amino acid profiles of all the varieties were similar to the composition of the Turkish hazelnut (Köksal et al., 2006).

**TABLE 3:** Free aminoacid content and hydrolyzed aminoacid content of hazelnut kernels from different Chinese hazelnut species and cultivars.

**Name of amino acid**			**Species/cultivars**				
			***C. kweichowensis***	***C. mandshurica***	***C. heterophylla***	***C. yunnanensis***	**cv. ‘Dawei’**
FAA (mg/100 g DW)	EAA	Thr	11.27 ± 0.18a	2.43 ± 0.97c	1.57 ± 0.00c	9.00 ± 0.18b	1.56 ± 0.00c
		Val	1.04 ± 0.00a	1.04 ± 0.00a	1.05 ± 0.00a	1.26 ± 0.36a	1.56 ± 0.00a
		Met	ND	ND	ND	0.36 ± 0.18	ND
		Lys	1.04 ± 0.00a	0.52 ± 0.00b	0.52 ± 0.00b	0.54 ± 0.00b	0.52 ± 0.00b
		Ile	0.69 ± 0.17a	0.69 ± 0.17a	0.52 ± 0.00a	0.72 ± 0.18a	0.52 ± 0.00a
		Leu	0.69 ± 0.17ab	0.69 ± 0.17ab	0.52 ± 0.00ab	0.90 ± 0.18a	0.35 ± 0.17b
		Phe	0.52 ± 0.00	0.52 ± 0.00	0.52 ± 0.00	0.54 ± 0.00	0.52 ± 0.00
		Trp	ND	ND	ND	ND	ND
		Total	15.26 ± 0.17a	5.91 ± 1.14c	4.71 ± 0.00c	13.32 ± 0.48b	5.03 ± 0.17c
	NEAA	Asp	2.43 ± 0.18a	1.04 ± 0.00b	2.27 ± 0.18a	2.34 ± 0.18a	2.60 ± 0.00a
		Ser	1.91 ± 0.17b	1.04 ± 0.00c	2.09 ± 0.00b	2.70 ± 0.00a	1.91 ± 0.17b
		Glu	4.68 ± 0.00d	4.34 ± 0.17d	6.10 ± 0.18c	6.84 ± 0.18b	8.15 ± 0.35a
		Gly	0.52 ± 0.00b	1.04 ± 0.00a	0.52 ± 0.00b	0.90 ± 0.18a	1.04 ± 0.00a
		His	0.52 ± 0.00b	ND	0.87 ± 0.18a	0.54 ± 0.00b	0.52 ± 0.00b
		Arg	ND	0.17 ± 0.17b	10.29 ± 0.76a	ND	1.21 ± 0.35b
		Ala	3.64 ± 0.00a	2.95 ± 0.35b	1.57 ± 0.00d	3.78 ± 0.00a	2.08 ± 0.00c
		Pro	2.60 ± 0.00a	2.78 ± 0.17a	1.92 ± 0.17b	1.98 ± 0.18b	1.04 ± 0.00c
		Tyr	0.87 ± 0.17a	0.69 ± 0.17a	0.52 ± 0.00a	0.90 ± 0.18a	0.87 ± 0.17a
		Total	17.17 ± 0.31c	14.07 ± 0.02d	26.15 ± 0.80a	19.98 ± 0.31b	19.42 ± 0.17b
	Total FAA		32.43 ± 0.36ab	19.98 ± 1.12c	30.86 ± 0.80b	33.29 ± 0.36a	24.45 ± 0.30d
HAA (g/100 g DW)	EAA	Thr	0.44 ± 0.03bc	0.41 ± 0.00c	0.65 ± 0.00a	0.48 ± 0.01b	0.43 ± 0.00c
		Val	0.57 ± 0.01b	0.48 ± 0.00c	0.74 ± 0.02a	0.51 ± 0.00c	0.50 ± 0.00c
		Met	0.11 ± 0.01b	0.07 ± 0.00b	0.08 ± 0.00b	0.40 ± 0.00a	0.11 ± 0.00b
		Lys	0.27 ± 0.01c	0.35 ± 0.00a	0.31 ± 0.00b	0.29 ± 0.01bc	0.29 ± 0.00bc
		Ile	0.53 ± 0.01b	0.46 ± 0.00c	0.69 ± 0.01a	0.47 ± 0.03c	0.45 ± 0.01c
		Leu	0.87 ± 0.02b	0.75 ± 0.01c	1.17 ± 0.03a	0.87 ± 0.02b	0.79 ± 0.01c
		Phe	0.82 ± 0.01b	0.62 ± 0.00e	1.17 ± 0.01a	0.72 ± 0.01c	0.69 ± 0.02d
		Trp	0.28 ± 0.00a	0.16 ± 0.01d	0.27 ± 0.00a	0.22 ± 0.00b	0.20 ± 0.00c
		Total	3.88 ± 0.03b	3.30 ± 0.01c	5.08 ± 0.05a	3.98 ± 0.18b	3.47 ± 0.02c
	NEAA	Asp	0.72 ± 0.02c	0.77 ± 0.01b	0.88 ± 0.01a	0.69 ± 0.00c	0.77 ± 0.01b
		Ser	0.60 ± 0.03b	0.53 ± 0.00c	0.79 ± 0.01a	0.56 ± 0.01b	0.53 ± 0.02c
		Glu	1.73 ± 0.05b	1.82 ± 0.03b	2.24 ± 0.04a	1.72 ± 0.02b	1.77 ± 0.04b
		Gly	0.69 ± 0.03b	0.61 ± 0.00c	0.96 ± 0.01a	0.71 ± 0.00b	0.63 ± 0.00c
		His	0.44 ± 0.01bc	0.37 ± 0.01d	0.65 ± 0.01a	0.45 ± 0.00b	0.41 ± 0.00c
		Arg	2.03 ± 0.03b	1.61 ± 0.02d	2.69 ± 0.02a	1.78 ± 0.02c	1.71 ± 0.03c
		Ala	0.43 ± 0.01c	0.45 ± 0.00b	0.57 ± 0.00a	0.44 ± 0.01bc	0.44 ± 0.00bc
		Pro	0.54 ± 0.01b	0.48 ± 0.00c	0.68 ± 0.03a	0.53 ± 0.01b	0.47 ± 0.01c
		Tyr	0.57 ± 0.00b	0.42 ± 0.05d	0.78 ± 0.04a	0.51 ± 0.00c	0.45 ± 0.00cd
		Total	7.74 ± 0.04b	7.07 ± 0.05d	10.25 ± 0.07a	7.38 ± 0.02c	7.18 ± 0.04d
	Total HAA		11.63 ± 0.07b	10.37 ± 0.06c	15.32 ± 0.10a	11.36 ± 0.19b	10.65 ± 0.04c

*Data are means ± standard error (SE), n = 3. Values in the same column with different letters are significantly different (p < 0.05). FAA, free amino acid; HAA, hydrate amino acid; EAA, necessary amino acid; NEAA, unnecessary; ND, not detected. Thr, Threonine; Val, Valine; Met, Methionine; Lys, Lysine; Ile, Isoleucine; Leu, Leucine; Phe, Phenylalanine; Trp, Tryptophane; Asp, Aspartic acid; Ser, Serine; Glu, Glutamic acid; Gly, Glycine; His, Hlstidine; Arg, Argnine; Ala, Alanine; Pro, Proline; Tyr, Tyrosine.*

In this study, the hydrolyzed amino acid (HAA) composition and the content of each hazelnut kernel is given in Table 3. Seventeen amino acids were detected in each hazelnut sample, including eight EAAs and nine NEAAs, with differences being obvious for each of them. The contents of the total EAA and the total NEAA were 3.30–5.08 and 7.07–10.25 g/100 g DW, respectively, and that of *C. heterophylla* was the highest value. All varieties of hazelnut had relatively high contents of Glu and Argnine (Arg), at 1.73–2.24 and 1.61–2.69 g/100 g DW, respectively. The total HAA content ranged from 10.37 to 15.32 g/100 g DW, and *C. heterophylla* had the highest total HAA content among these five hazelnut kernel samples. The total HAA content of the hybrid hazelnut cv. ‘Dawei’ was lower than that of the female parent *C. heterophylla* but was higher than that of the other three wild hazelnut species.

### Sugar Composition

**FIGURE 2:**
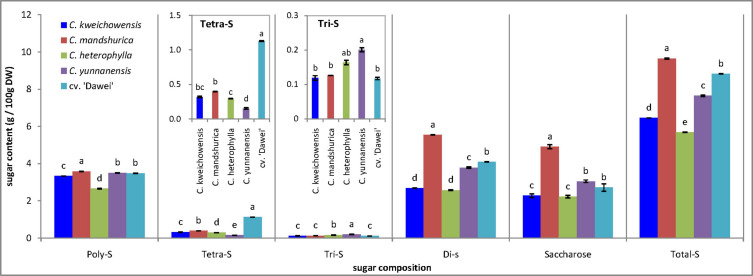
Sugar content of hazelnut kernels from different Chinese hazelnut species and cultivars. Poly-S, polysaccharide; Tetra-S, tetrasaccharide; Tri-S, trisaccharide; Dis-S, disaccharide; Total-S, total sugar. Data are presented as means ± SE (standard error) of three independent experiments with three replicates for each. Values followed by a different letter were significantly different according to Duncan’s multiple range test at *P <* 0.05.

Generally, the sugar content of the hazelnut kernel is not considered and has been seldom reported. In previous research, the total sugar content ranged from 12.66 to 19.09 g/100 g FW among 33 hybrid hazelnut (*Corylus heterophylla* Fisch. × *C. avellana* L.) cultivars, and the average total sugar content was 12.49 g/100 g FW in *C. heterophylla* (Tian et al., 2012). Wang reported that the carbohydrates of five cultivars of hazelnut from United States were approximately 16–20 g/100 g (Wang et al., 2018). 24 cultivars from Italian were reported that the content of sugar ranged from 3.98 to 5.95 g/100 g (Cristofori et al., 2008). The present study showed that the total sugar content was between 5.69 g/100 g DW (*C. heterophylla*) and 9.65 g/100 g DW (*C. mandshurica*) in the five samples of hazelnut considered (Figure 2). As a new hazelnut species, the hybrid hazelnut cv. ‘Dawei’ had a remarkably increased sugar content compared to the female parent *C. heterophylla*. The highest levels of polysaccharides (3.58 g/100 g DW), tetrasaccharides (1.12 g/100 g DW), trisaccharides (0.20 g/100 g DW), and disaccharides (5.53 g/100 g DW), as the predominant sugars, were determined in the kernels of *C. mandshurica*, the hybrid hazelnut cv. ‘Dawei’, *C. yunnanensis*, and *C. mandshurica*, respectively. One of the most important results was that the contents of polysaccharide, tetrasaccharide, disaccharide sharply increased, and trisaccharide dramatically decreased in the hybrid hazelnut cv. ‘Dawei’ compared to *C. heterophylla*, but the saccharose contents of the two hazelnut kernels were the same. Saccharose was the most important and abundant disaccharide in these hazelnut kernel samples, ranging from 2.20 to 4.99 g/100 g DW. Xylose, galactose, mannitose, glucose, and fructose were not detected in these hazelnut kernel samples.

### Content of Vitamin C and Tocopherol

As is well known, vitamin C is an important antioxidant (Halliwell, 1996). Generally, vitamin C, as a water-soluble vitamin, has a level less than 10 mg/100 g in nuts compared to the high vitamin C content of most fruits, such as kiwifruit, jujube and orange at over 100 mg/100 g and strawberry, tomato and longan at over 50 mg/100 g (Ma and Liu, 2011). Vitamin C content was determined to be between 10.24 and 16.68 mg/100 g DW in the five hazelnut kernel samples (Table 4). *C. yunnanensis* was the most significant species based on vitamin C content, while the other four hazelnut kernel samples had the same levels.

**TABLE 4:** Vitamin C, tocopherol, total phenol and total flavonoid of hazelnut kernels from different Chinese hazelnut species and cultivars.

**Species/cultivars**	**Vc content (mg/100 g DW)**	**tocopherol content (mg/100 g oil)**				**TPC (mg GAE/100 g DW)**	**TFC (mg/100 g DW)**
		**α-T**	**(β+γ)-T**	**δ-T**	**Total-T**		
*C. kweichowensis*	11.53 ± 0.40b	19.72 ± 0.40d	1.14 ± 0.08e	0.74 ± 0.12	21.60 ± 0.45c	160.61 ± 0.68b	190.53 ± 2.11c
*C. mandshurica*	10.24 ± 0.14c	24.31 ± 0.40bc	15.12 ± 0.02a	ND^*b*^	39.43 ± 0.39a	120.82 ± 3.10d	174.29 ± 4.30c
*C. heterophylla*	11.50 ± 0.38b	23.54 ± 0.73c	6.21 ± 0.04c	ND^*b*^	29.75 ± 0.70b	141.23 ± 1.83c	252.82 ± 13.69b
*C. yunnanensis*	16.85 ± 0.26a	27.03 ± 1.04a	11.23 ± 0.13b	ND^*b*^	38.26 ± 1.16a	205.82 ± 0.83a	356.90 ± 4.41a
cv. ‘Dawei’	10.50 ± 0.47bc	26.23 ± 0.93ab	2.09 ± 0.07d	ND^*b*^	28.32 ± 0.98b	66.53 ± 3.55e	80.46 ± 2.02d

*Data are means ± standard error (SE), n = 3. Values in the same column with different letters are significantly different (p < 0.05). Vc, vitamin C; α—T, α—tocopherol; (β+γ)—T, (β+γ)—tocopherol; δ—T, δ—tocopherol; total—T, total tocopherol; TPC, total phenol content; TFC, total flavonoid content; ND, not detected.*

Vitamin E, another name tocopherol, as a vital component required for reproduction, was discovered in 1922, and eight naturally occurring vitamin E isoforms, namely, α-, β-, γ-, and δ-tocopherol and α-, β-, γ-, and δ-tocotrienol, have been confirmed. Vitamin E is a potent antioxidant, as a particularly important functional component in foods, widely exists in nuts, such as walnuts, pecans, almonds, and cashews (Peh et al., 2016). In a former report, three types of tocopherols α-, β-, γ-, and α-tocotrienol were detected in hazelnut kernel oil collected from Turky and Germany, the total tocopherol content was 25.8–69.8 mg/100 g oil and α-tocopherol was the main component from 19.9 to 63.9 mg/100 g (Matthäus and Özcan, 2012). Other two reports showed that 14 Turkish varieties hazelnut kernels and four American varieties hazelnut kernels were the similar type (α-, β-, and γ-) but different content of tocopherols (Taş and Gökmen, 2015; Wang et al., 2018). Interestingly, a similar result that α-tocopherol was the most abundant tocopherol, accounting for 90–92% of the total tocopherol content, was reported in a study of Polish hazelnut kernel samples (Ciemniewska-Zytkiewicz et al., 2015). In this study (Table 4), the main α-tocopherol content level of all five hazelnut kernel samples widely ranged from 19.72 to 27.03 mg/100 g oil. (β+γ)-tocopherol contents were remarkably different among the five hazelnut kernel samples, ranging from 1.14 to 15.12 mg/100 g oil. δ-tocopherol was detected only in *C. kweichowensis*, at a very low level of 0.74 mg/100 g oil. The total tocopherol content ranged between 21.60 and 39.43 mg/100 g oil in this research, which was similar to that of former reports (Kornsteiner et al., 2006; Wang et al., 2018). *C. mandshurica* and *C. yunnanensis* had similar tocopherol profiles, with obviously higher total tocopherol content than that of the other hazelnut kernels. Compared to the female parent *C. heterophylla*, the hybrid hazelnut cv. ‘Dawei’ kernel had higher α- tocopherol content, lower (β+γ)- tocopherol content and higher total tocopherol content.

### Total Phenol Content (TPC) and Total Flavonoid Content (TFC)

Phenolic compounds range structurally from a simple phenolic molecule to complex high-molecular-weight polymers and are the primary bioactive components of plants. Phenolic components are an essential part of the human diet and are of considerable interest due to their antioxidant properties and potential beneficial health effects. More and more evidence indicates that the consumption of a variety of phenolic compounds present in foods may lower the risk of health disorders because of their antioxidant activity (Locatelli et al., 2010; Altun et al., 2013; Shahidi and Ambigaipalan, 2015; Taş and Gökmen, 2015). So far, 23 compounds from different phenolic groups were detected, and 15 of them were identified from raw hazelnut kernels (Jakopic et al., 2011), and 22 phenolic compounds were identified, 16 of which were identified for the first time in roasted hazelnut kernels, including flavonoids, phenolic acids and related compounds, hydrolysable tannins and related compounds, and other phenolics (Pelvan et al., 2018). Some study reported that the total phenolic content of natural Turkish ‘Tombul’ hazelnut kernels was 171 mg GAE/100 g DW (Pelvan et al., 2018) and that the varieties from Slovenia ranged from 49.10 to 170.04 mg GAE/100 g (Slatnar et al., 2014). The present study shows that the total phenol and flavonoid contents ranged widely, from 66.53 to 205.82 mg GAE/100 g DW and from 80.46 to 356.90 mg/100 g DW, respectively (Table 4). *C. yunnanensis* had the highest total phenol and flavonoid contents, while *C. mandshurica* had the lowest content among the four wild hazelnut kernel samples. It should be noted that the total phenol and flavonoid contents all decreased sharply compared to the female parent *C. heterophylla*, dropping from 141.23 to 66.53 mg GAE/100 g DW and from 252.82 to 80.46 mg/100 g DW, respectively. This result was in accordance with reports from Ghirardello (Kornsteiner et al., 2006).

### Antioxidant Abilities of Kernel and Oil

**FIGURE 3:**
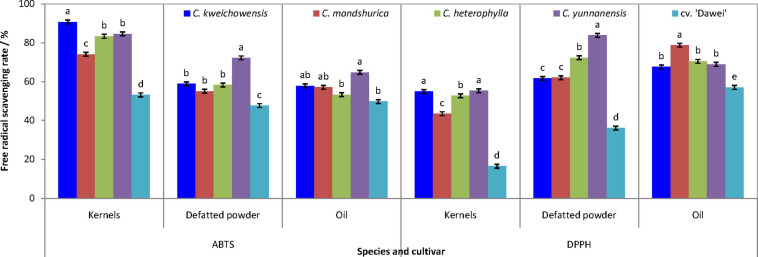
Scavenging rate of ABTS and DPPH free radical of hazelnut kernels, defatted powder and oil from different Chinese hazelnut species and cultivars. Data are presented as means ± SE (standard error) of three independent experiments with three replicates for each. Values followed by a different letter were significantly different according to Duncan’s multiple range test at *P <* 0.05.

The antioxidant abilities of hazelnut kernels were measured as the free radical scavenging rate (FRSR) of DPPH (2,2-diphenyl-1-picrylhydrazyl) and ABTS (2,2-azinobis (3-ethyl-benzothiazoline-6-sulfonic acid)). As indicated in Figure 3, the tendency of the two FRSRs was very similar among the kernel, the defatted flour and the oil in each hazelnut. High FRSR was the common and obvious characteristic for wild hazelnut kernels compared to the hybrid hazelnut cv. ‘Dawei’. In terms of the four wild hazelnut kernels, the FRSR of *C. mandshurica* kernel was lower than that of the other three hazelnut kernels, and the defatted flour of *C. yunnanensis* kernel had the highest FRSR. *C. mandshurica* and *C. yunnanensis* oils had the highest FRSR of ABTS and DPPH, respectively. Thus, it can be seen that the antioxidant ability of wild hazelnut kernels was remarkably higher than that of the hybrid hazelnut cv. ‘Dawei’.

Previous studies indicated that changes in the trolox equivalent antioxidant capacity (TEAC) and the radical scavenging activity (RSA) values followed a pattern similar to that of TPC during hazelnut storage (Ghirardello et al., 2016). Further analysis showed that there was the same trend between FRSR of ABTS and DPPH for all the samples of kernel and defatted flour. Two ABTS of FRSR between defatted flours and oils also followed a similar trend. The correlation analysis indicated (Table 5) that the FRSR of ABTS and DPPH of defatted flour, the ABTS of oil and DPPH of kernel all had a positive correlation with TPC (the correlation coefficients were 0.969, 0.926, 0.913, and 0.906, respectively). The FRSR of ABTS and DPPH of defatted flour, the DPPH of kernel were positively correlated with TFC (with correlation coefficients of 0.967, 0.970, and 0.803, respectively). The FRSR of ABTS of defatted powder and oil were positively correlated with vitamin C (with correlation coefficients of 0.924 and 0.844, respectively). Previous studies have verified that the high antioxidant activity of hazelnut was related to higher phenolic content (Ghirardello et al., 2016; Pelvan et al., 2018; Wang et al., 2018), thus, it can be seen that higher antioxidant capacity could be due to the presence of condensed phenolics. The same result was indicated in hazelnut by Yuan’s report (Yuan et al., 2018) and Shahidi report (Shahidi et al., 2007). But, vitamin C is a water-soluble substance, so there is a significant correlation between vitamin C content in the free radical scavenging ability of defatted powder. Although hazelnut is rich in oil, the function of water-soluble antioxidant components (such as vitamin C) in hazelnut cannot be ignored.

**TABLE 5:** Correlation between the nutrient substance and free radical scavenging ability from different hazelnut kernel product.

		**ABTS**	**DPPH**
Defatted Powder	TPC	0.969**	0.926**
	TFC	0.967**	0.970**
	Vc	0.924**	0.736
	Total—T	–	–
Oil	TPC	0.913**	0.412
	TFC	0.828*	0.402
	Vc	0.844*	0.055
	Total—T	0.380	0.098
Kernels	TPC	0.862**	0.906**
	TFC	0.717	0.803*
	Vc	0.412	0.500
	Total–T	−0.163	0.001

**Correlation is significant at the 0.01 level (2-tailed). ^∗^Correlation is significant at the 0.05 level (2-tailed). TPC, total phenol content; TFC, total flavonoid content; Vc, vitamin C; total—T, total tocopherol; ABTS, 2,2-azinobis (3-ethyl-benzothiazoline-6-sulfonic acid); DPPH, 2,2-diphenyl-1-picrylhydrazyl.

## Conclusion

The oil content and fatty acid composition of hazelnut kernels are different among species. Compared to the wild species and the hybrid hazelnut (*Corylus. Heterophylla* Fisch. × *Corylus. avellana* L.) cv. ‘Dawei’ kernels, *C. heterophylla* has obvious characteristics of low oil content and high linoleic acid content. The hybrid hazelnut cv. ‘Dawei’, has higher oil content than the four wild species, and maintains the characteristics of high UFA content similar to its female parent *Corylus heterophylla*; However, the ratio of MUFA/PUFA is completely different from that of its female parent. The hybrid hazelnut cv. ‘Dawei’ has the characteristics of high oleic acid and low linoleic acid. Whether this characteristic comes from its male Italian parent needs further study. In these five hazelnuts, Tryptophan content in free amino acids of hazelnut kernels is very low and hardly be detected, the composition and content of hydrolyzed amino acids are quite different among species. Sugar in hazelnut kernels is mainly sucrose. Compared with other species, *C. mandshurica* has obvious characteristics of high sucrose content. The wild species hazelnut kernels contain more bioactive components such as vitamin C, tocopherols, polyphenols and flavonoids, which may help it overcome the harsh growth environment.

Overall, there were great differences in the nutritional composition of different hazelnut species. Wild species are a good source of breeding materials because of their own characteristics in nutrition composition, and hybrid hazelnut cv. ‘Dawei’ with good quality has the value of commercial promotion.

## Data Availability Statement

The original contributions presented in the study are included in the article/supplementary material, further inquiries can be directed to the corresponding author/s.

## Author Contributions

JJ organized the database, performed the statistical analysis, and wrote sections of the manuscript. LL wrote the first draft of the manuscript. All authors contributed to manuscript revision, read, and approved the submitted version, and contributed to conception and design of the study.

## Conflict of Interest

The authors declare that the research was conducted in the absence of any commercial or financial relationships that could be construed as a potential conflict of interest.

## Abbreviations

DW: dry weight
GC: gas chromatography
FID: flame ionization detection
FAMD: fatty acid methyl ester
RP-HPLC: reversed-phase high performance liquid chromatography fluorescence detection
Ex: excitation wavelength
Em: emission wavelength
HPLC: high performance liquid chromatography
RID: refractive index detector
FRSR: free radical scavenging rate
DPPH: 2,2-diphenyl-1-picrylhydrazyl
ABTS: 2,2-azinobis (3-ethyl-benzothiazoline-6-sulfonic acid)
UFA: unsaturated fatty acids
SFA: saturated fatty acids
MUFA: monounsaturated fatty acid
PUFA: polyunsaturated fatty acid
FAME: fatty acid methyl ester
TPrC: total protein content
SPrC: soluble protein content
FAA: free amino acid
EAA: essential amino acid
NEAA: non-essential amino acid
HAA: hydrolyzed amino acid
TPC: total phenol content
TFC: total flavonoid content
GAE: gallic acid equivalent
TEAC: trolox equivalent antioxidant capacity
RSA: radical scavenging activity.
